# The Phytoalexin Resveratrol Regulates the Initiation of Hypersensitive Cell Death in *Vitis* Cell

**DOI:** 10.1371/journal.pone.0026405

**Published:** 2011-10-28

**Authors:** Xiaoli Chang, Ernst Heene, Fei Qiao, Peter Nick

**Affiliations:** 1 Department of Molecular Cell Biology, Botanical Institute 1, Karlsruhe Institute of Technology, Karlsruhe, Germany; 2 Institute of Tropical Crops Genetic Resources, Chinese Academy of Tropical Agricultural Sciences, Danzhou, China; Roswell Park Cancer Institute, United States of America

## Abstract

Resveratrol is a major phytoalexin produced by plants in response to various stresses and promotes disease resistance. The resistance of North American grapevine *Vitis rupestris* is correlated with a hypersensitive reaction (HR), while susceptible European *Vitis vinifera* cv. ‘Pinot Noir’ does not exhibit HR, but expresses basal defence. We have shown previously that in cell lines derived from the two *Vitis* species, the bacterial effector Harpin induced a rapid and sensitive accumulation of *stilbene synthase* (*StSy*) transcripts, followed by massive cell death in *V. rupestris*. In the present work, we analysed the function of the phytoalexin resveratrol, the product of StSy. We found that cv. ‘Pinot Noir’ accumulated low resveratrol and its glycoside *trans*-piceid, whereas *V. rupestris* produced massive *trans*-resveratrol and the toxic oxidative δ-viniferin, indicating that the preferred metabolitism of resveratrol plays role in *Vitis* resistance. Cellular responses to resveratrol included rapid alkalinisation, accumulation of *pathogenesis-related protein* 5 (*PR*5) transcripts, oxidative burst, actin bundling, and cell death. Microtubule disruption and induction of *StSy* were triggered by Harpin, but not by resveratrol. Whereas most responses proceeded with different amplitude for the two cell lines, the accumulation of resveratrol, and the competence for resveratrol-induced oxidative burst differed in quality. The data lead to a model, where resveratrol, in addition to its classical role as antimicrobial phytoalexin, represents an important regulator for initiation of HR-related cell death.

## Introduction

Grapevine, an economically and culturally important crop, is affected by a wide range of pathogens, causing yield losses and impairing wine quality. During a long history of coevolution between host and pathogens such as Downy and Powdery Mildew, North American *Vitis* species have developed defence mechanisms based on specific host receptors capable to activate defence after recognition of pathogen effectors (effector-triggered immunity, ETI) [1], often culminating in a hypersensitive reaction (HR). HR is a plant-specific form of programmed cell death (PCD) associated with plant resistance to pathogen infection and characterized by a rapid and localized death of tissues at the site of infection to limit further pathogen multiplication and spread [2], [3]. Since Downy and Powdery Mildew arrived in Europe only in 1869, ETI against these pathogens is absent in the cultivated grapevine *Vitis vinifera*. However, so called basal defence, effective against a broad variety of pathogen strains, but not culminating in HR, is active in grapevine. Although triggered by different receptors, both responses share molecular components and cellular mechanisms, and differ on the quantitative rather than on the qualitative level [4].

Phytoalexins, a functionally defined class of secondary metabolites, are generated *de novo* in response to stress factors such as pathogen attack. In grapevine, stilbenes, in general, and resveratrol (*trans*-3, 4′, 5-trihydroxystilbene) in particular, are well-known phytoalexins active against Downy and Powdery Mildew. Transcription of *stilbene synthases* (*StSy*), key enzymes for resveratrol synthesis, is induced by pathogens, activation of the jasmonate pathway [5], or abiotic stresses [6]. Engineering the *StSy* into plants of interest results in resveratrol accumulation and elevates pathogen resistance in some crops such as rice [7], tomato [8], or barley [9]. Stilbene synthases are typically organised in gene families with high sequence homology of individual members, but different regulatory features in their promotors [10]. For the sake of simplicity, in this study, the term stilbene synthase is used to designate this family of enzymes. Resveratrol acts as a precursor for stilbene compounds of higher fungitoxicity that accumulate in grapevine as a result of infection or stress [11]. Among those, especially δ-viniferin inhibits zoospores mobility of Downy Mildew (*Plasmopara viticola*), whereas the glycoside piceid is not active [12], [13].

In addition to its antimicrobial activity, resveratrol has also attracted attention based on its health benefits to human including prevention of cardiovascular disease, protection against cancers, obesity, diabetes and neurodegenerative diseases, and extension of lifespan by mimicking the caloric restriction effect [14], [15], [16], [17]. A famous phenomenon is termed as “French Paradox” which describes that mild consumption of red wine can reduce the risk of heart disease due to resveratrol content in the red wine [18].

North American *Vitis rupestris* has evolved sympatrically with several of the major grapevine diseases, and can counteract pathogen attack not only by induction of phytoalexins, but, in addition, initiation of HR [2]. HR is not only triggered by pathogens, but also by the Harpin elicitor, a type-III bacterial effector derived from *Erwinia amylovora*, the causative agent of fire blight in Rosaceae [19]. In non-host plants, using Harpin, defence responses in two grapevine cell lines derived from the pathogen-sensitive cultivar ‘Pinot Noir’ and the pathogen-resistant *V. rupestris* were compared [20]. *V. rupestris* readily responded to Harpin with a massive HR-type of cell death occurring within 48 h [21], and showed a strong, rapid, and transient accumulation of *StSy* transcripts. This response was accompanied by disruption of cortical microtubules, and massive bundling of actin filaments. Pharmacological manipulation of microtubules enhanced accumulation of *StSy* transcripts in the absence of elicitor [20]. Conversely, the responses in cv. ‘Pinot Noir’ were weaker. This leads to the question – at what point these quantitative differences are transformed into a qualitatively different output (basal defence versus HR-mediated cell death)? The cellular effects of resveratrol on fungi have been investigated in some studies [22], [23]. However, to our knowledge, the resveratrol responses of the phytoalexin-producing plant cells themselves have not been addressed previously.

In this study, we show that, in response to Harpin, the pathogen sensitive cv. ‘Pinot Noir’ produces low resveratrol and its glycoside piceid, whereas the resistant *V. rupestris* trends to accumulate abundant resveratrol and the potent oxidised dimmer δ-viniferin. Exogenous resveratrol inhibits cell growth in a dose-dependent manner and activates defence-related responses such as rapid alkalinisation, and accumulation of transcripts for the *pathogen-related proteins* 5 and 10 (*PR*5 and *PR*10), a mild elevation of ROS-formation, actin bundling, and cell death. Unlike Harpin, resveratrol does not induce transcripts for *RS* and *StSy*, nor does it affect microtubule structure. In *V. rupestris*, Harpin induces rapid and massive formation of ROS, and suppression of production and/or scavenging of apoplastic ROS impairs the elicitor-induced accumulation of *StSy* transcripts. The data are interpreted by a model, where resveratrol, in addition to its classical function as antimicrobial phytoalexin, acts as a signaling molecule in the regulation of the initiation of HR-related cell death.

## Results

### Resveratrol production is triggered by the Harpin elicitor

**Figure 1:**
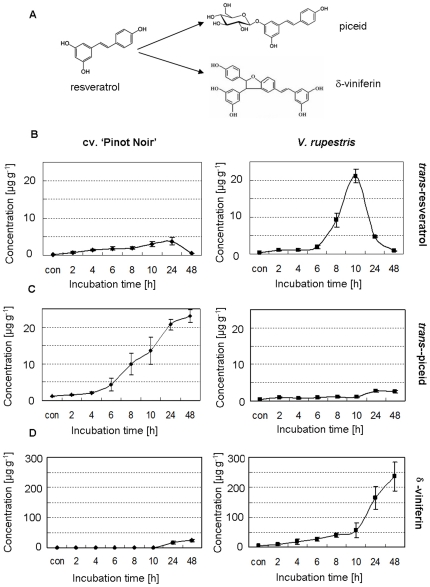
Accumulation of stilbenes in response to Harpin in cv. ‘Pinot noir’ and *V. rupestris*. **A** Alternative pathways for the stilbenic resveratrol by glycosylation leading to *trans*-piceid or oxidation leading to δ-viniferin. Time courses for the accumulation of *trans*-resveratrol (**B**), *trans*-piceid (**C**), and δ-viniferin (**D**) after treatment with 9 µg l^−1^ Harpin are plotted as mean values and standard errors from at least five independent experimental series.

The Harpin elicitor induced a transient accumulation of *stilbene synthase* (*StSy*) transcripts, which was strong in *V. rupestris* as compared to *V. vinifera* cv. ‘Pinot noir’ [20]. To investigate, whether the product of StSy, i.e. the stilbenic resveratrol, or its derivatives (Figure 1A), also accumulates in response to the elicitor. The abundance of resveratrol was quantified by HPLC in both cell lines after Harpin treatment.

*Trans*-resveratrol was detected from 2 h in both cell lines. However, in *V. rupestris*, the amount of *trans*-resveratrol increased sharply from 6 h after elicitation, reaching a maximum of more than 21.1 µg g^−1^ f.w. at 10 h (corresponding to >90 µM), followed by a sharp decline. In cv. ‘Pinot noir’, the response was weaker and later with a maximal induction of 3.8 µg g^−1^ f.w. (around 15 µM) at 24 h after elicitation (Figure 1B).

In addition to *trans*-resveratrol, its metabolic products, the glycoside *trans*-piceid and the oxidised dimer δ-viniferin, were followed over time. *Trans*-piceid increased dramatically from 6 h, and reached 23.1 µg g^−1^ f.w. at 48 h (corresponding to >60 µM) in cv. ‘Pinot noir’ (Figure 1C), even during the later stages, when *trans*-resveratrol decreased (compare Figures 1B, C), indicating rapid glycosylation of the *trans*-resveratrol might produce in response to Harpin. In *V. rupestris*, *trans*-piceid increased more slowly and to a much lower level (only 1/10 of that reached in cv. ‘Pinot Noir’).

In contrast to piceid, Harpin strongly induced δ-viniferin in *V. rupestris* (Figure 1D). The increase of δ-viniferin was first slow, but proceeded steadily. From 10 h, the accumulation was accelerated reaching 236.2 µg g^−1^ f.w. (corresponding to 450 µM) at 48 h. Thus, the bulk of δ-viniferin accumulation coincides with the decline of its precursor resveratrol. In cv. ‘Pinot Noir’, δ-viniferin accumulated only to 23.5 µg g^−1^ f.w.

### Resveratrol responses of cell growth and rapid alkalinisation

**Figure 2:**
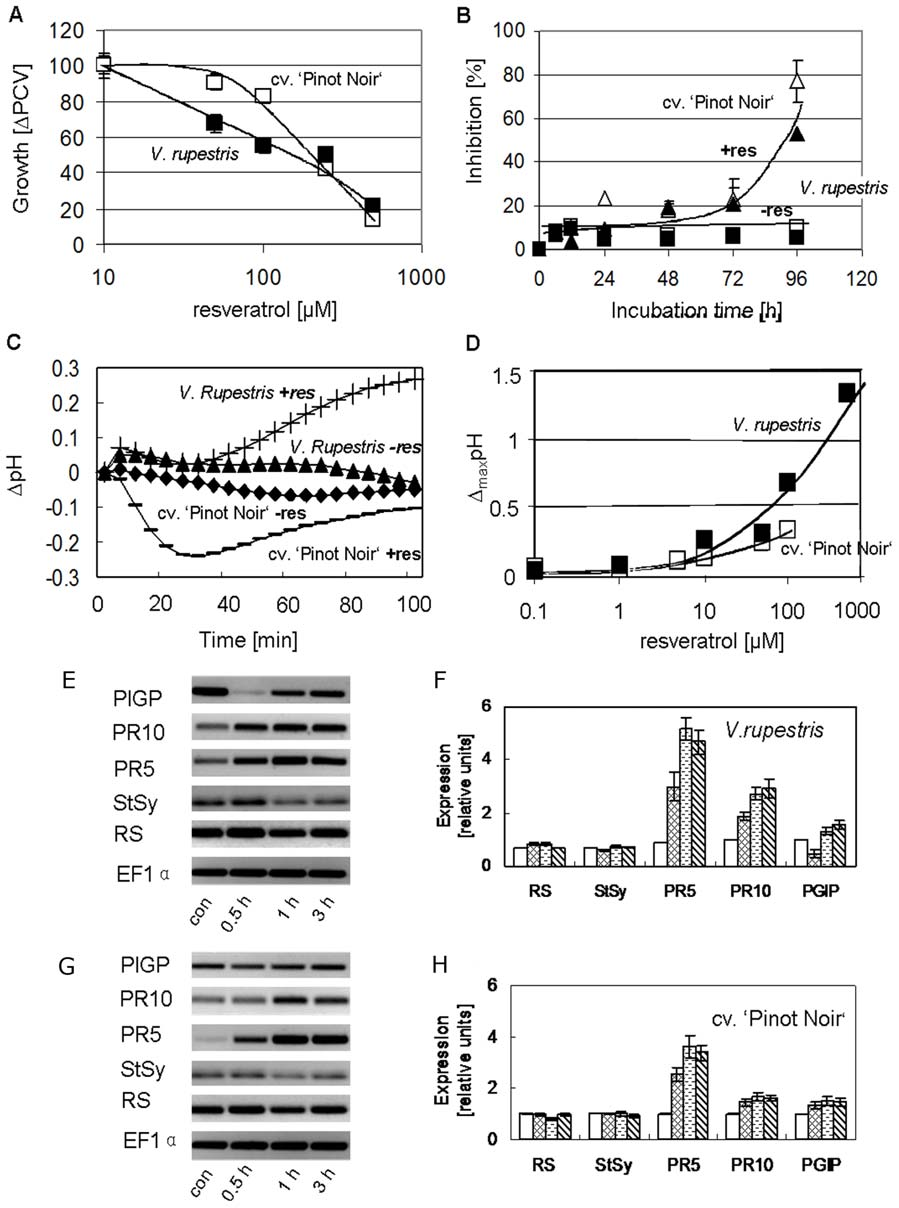
Responses of cv. ‘Pinot noir’ and *V. rupestris* to resveratrol. **A** Dose-response relation for growth (measured as increment in packed cell volume) over resveratrol concentration. Data show means from four independent experimental series. **B** Time course of growth inhibition in response to 50 µM resveratrol as compared to the solvent control (equal concentration of ethanol), values show means from four independent experimental series, bars standard errors. **C** Representative time course of extracellular alkalinisation induced by 50 µM resveratrol (+res) versus the solvent control (−res). **D** Dose-response relation for the steady-state response of pH over resveratrol concentration (assessed two hours after addition of resveratrol) **E–H** Response of defence-related genes to 50 µM resveratrol, including encoding reveratrol synthase (RS), stilbene synthase (StSy), pathogenesis-related protein genes 5 and 10 (PR5, PR10), and polygalacturonase inhibiting protein gene (PGIP) detected by semiquantitative RT-PCR. **E**,**G** shows a representative gel, **F**,**H** shows mean values and standard errors at con (control, white bars), 0.5 h (cross-hatched bars), 1 h (horizontally striped bars), and 3 h (boldly striped bars) after addition of 50 µM resveratrol from at least three independent experimental series, relative to the respective control value using elongation factor 1-α (EF1α) as internal standard.

To understand the biological function of the accumulation of resveratrol, we further investigated the cellular responses to exogenous resveratrol. Packed cell volume (PCV) at different concentrations of resveratrol was measured at the stationary phase after 7 days of growth (Figure 2A), and was found to decline from 10 µM resveratrol approached zero levels for 500 µM in both cell lines with a significantly stronger influence on *V. rupestris* as compared to cv. ‘Pinot Noir’. The time course of growth inhibition (Figure 2B) showed a lag of 48 h and reached 80% (as compared to the solvent control) at 96 hours in both cell lines. However, the inhibition was much more pronounced at 96 h compared to the stationary phase three days later (see the 50 µM values in Figure 2A). Thus, the inhibition was compensated with time, apparently more rapidly in cv. ‘Pinot Noir’ than in *V. rupestris*.

In the next step, extracellular alkalinisation, as a fast and convenient cellular indicator of plant defence [24], was analysed in response to 50 µM resveratrol (corresponding to the half-maximal concentration in response to the elicitor). In both cell lines, alkalinisation became detectable from 30 min, but developed more rapidly in *V. rupestris* (Figure 2C). The dose-response of steady-state pH (Figure 2D) showed an increase with rising concentrations of resveratrol reaching a maximal value of 1.25 units (similar to the maximal response achieved by Harpin elicitation as reported in Qiao *et al.* [20]. Although this increase presented as well, it was not as pronounced in cv. ‘Pinot Noir’. However, a reliable 500 µM point could not be measured for this cell line, because most cells collapsed leading to uncontrolled fluctuations of pH in consequence of vacuolar breakdown and cell death.

### Resveratrol-mediated defence-gene expression

To investigate whether exogenous resveratrol is able to activate defence genes, we followed the expression of defence genes encoding reveratrol synthase (RS), stilbene synthase (StSy), osmotin-type pathogenesis-related protein 5 (PR5), a member of the pathogenesis protein 10 (PR10) family, and polygalacturonase inhibiting protein (PGIP), which have been used previously for the Harpin response in *Vitis* cells [20]. For both cell lines, only minor fluctuations were observed for *PGIP* expression (Figure 2E–H). In contrast, transcripts for *PR*10 and, especially, *PR*5 increased rapidly and significantly from 30 min after addition of resveratrol. In *V. rupestris* (Figure 2E, F), the accumulation was much faster and to higher levels as compared to cv. ‘Pinot Noir’ (Figure 2G, H). It should be noted that *RS* and *StSy* transcripts that accumulated rapidly in response to Harpin [20], did not show a significant response to resveratrol.

### Resveratrol and Harpin induce reactive oxygen species differentially

**Figure 3:**
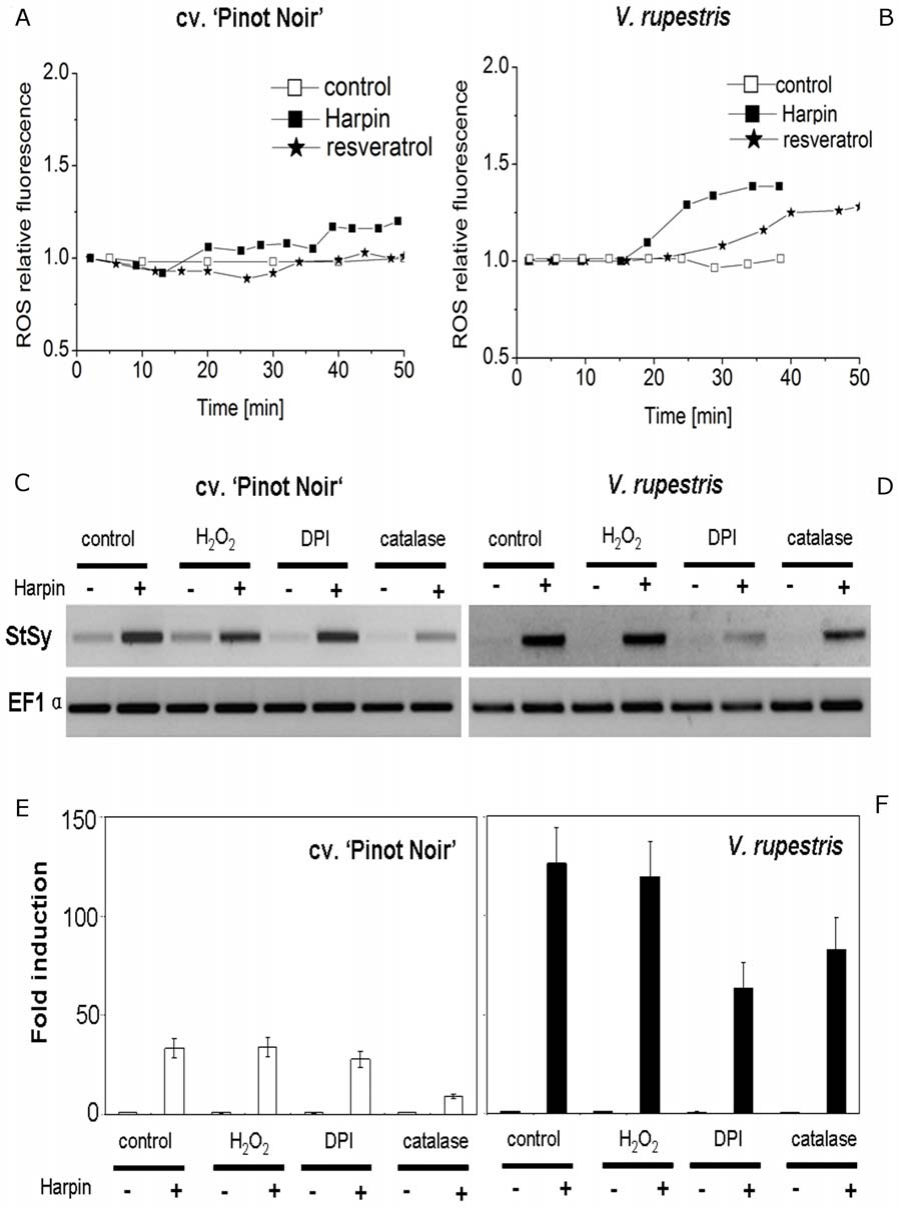
Production of reactive oxygen species (ROS) and effects on *stilbene synthase* (*StSy*) transcripts. **A, B** Time course accumulation of ROS using dihydrorhodamine 123 in response to the solvent control, Harpin (9 µg l^−1^), or resveratrol (50 µM). ROS relative fluorenscence was quantified relative to the respective base fluorescence by Image J software. **C–F** Effect of ROS on *StSy* expression to Harpin as assessed by RT-PCR for cv. ‘Pinot Noir’ (**C, E**) and *V. rupestris* (**D, F**). **C, D** Representative Gels for *StSy* transcripts 2 h after addition of Harpin (9 µg l^−1^), H_2_O_2_ (10 µM), Harpin with H_2_O_2_, NADPH oxidase inhibitor DPI (10 µM), Harpin with DPI, catalase (100 U) or Harpin with catalase. Water was added and used as control. **E, F** Mean values and standard errors from at least three independent experimental series, relative to the respective control value using elongation factor 1-α (EF1α) as internal standard.

Generation of reactive oxygen species (ROS) is associated with hypersensitive cell death [25]. To test, whether the elevated growth inhibition of the *V. rupestris* cell line (Figure 2B) is related to a facilitated induction of hypersensitive cell death, we used the fluorescent dye dihydrorhodamine 123 (DHR 123) to follow ROS production in response to either Harpin (9 µg l^−1^), or resveratrol (50 µM) compared to a solvent control. No significant changes were observed for the solvent control in two cell lines (Figure 3A, B). This basal fluorescence was elevated at 15 minutes after Harpin treatment in both cell lines. In *V. rupestris*, a further increase (about 1.5 folds) was observed from about 30–35 min after elicitation, but not obvious in cv. ‘Pinot Noir’. Application of 50 µM resveratrol nearly did not induce any increase of fluorescence in cv. ‘Pinot Noir’. In *V. rupestris*, the signal did increase, however, only distinct at around 40 min, i.e. later than in response to the Harpin elicitor.

### Are reactive oxygen species necessary for elicitor-triggered induction of *stilbene synthase*?

To test, whether the ROS triggered by the Harpin elicitor are necessary for the induction of *StSy*, we used gain- or loss-of-function experiments employing H_2_O_2_ as ROS-donor, whereas the NADPH oxidase inhibitor DPI, or the ROS-scavenger catalase were used to quell the increase of ROS abundance following challenge with Harpin. Analysis by semiquantitative RT-PCR showed that exogenous H_2_O_2_ failed to induce accumulation of *StSy* transcripts in absence of elicitor, nor could it amplify the response to Harpin (Figure 3C–F). However, application of DPI significantly suppressed the transcripts of *StSy* in both cell lines, but this inhibition was much more pronounced in *V. rupestris* than that in cv. ‘Pinot Noir’ (Figure 3E, F). Catalase inhibited *StSy* transcripts as well. However, in cv. ‘Pinot Noir’, inhibition by catalase was more efficient than by DPI, whereas this relation was reversed in *V. rupestris*. As to be expected, neither DPI nor catalase or H_2_O_2_ did induce any accumulation of *StSy* transcripts in absence of the elicitor. These results suggest that ROS are necessary for the induction of *StSy* transcripts in response to the Harpin elicitor. However, they are not sufficient to trigger *StSy* transcripts in the absence of the elicitor.

### Resveratol and Harpin induce cytoskeletal response differentially

A second aspect of hypersensitive-related programmed cell death is the reorganisation of actin [25], [26]. We have found in a previous study [20] that the cytoskeleton was reorganised in *Vitis* cells in response to the Harpin elicitor. We therefore compared the elicitor responses of microtubules and actin filaments to the responses to resveratrol.

**Figure 4:**
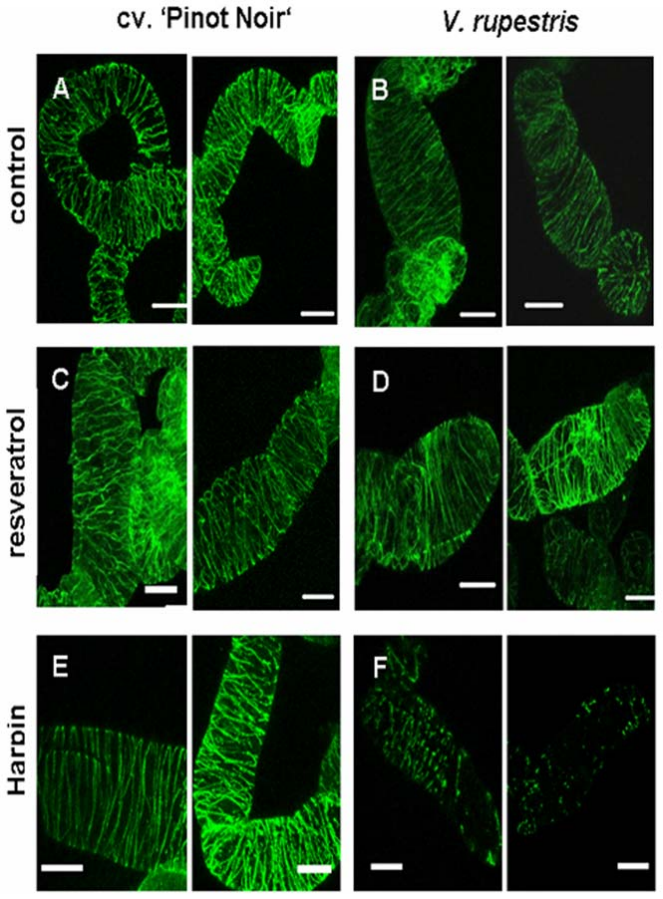
Responses of microtubules to Harpin and resveratrol. Cells of cv. ‘Pinot Noir’ (**A, C, E**) and *V. rupestris* (**B, D, F**) were treated with either ethanol as solvent control, with resveratrol (50 µM), or with Harpin (9 µg l^−1^), and microtubules were stained by means of immunofluorescence. Representative geometrical projections of confocal z-stacks are shown. Size bars = 20 µm.

When microtubules were visualised 30 min after treatment with either the solvent (Figure 4A, B), or with 50 µM resveratrol (Figure 4C, D), microtubules were not found to be different from untreated cells. However, treatment with Harpin (9 µg.l^−1^) led to disintegration of microtubules in *V. rupestris* but not in cv. ‘Pinot Noir’ (Figure 4E, F).

**Figure 5:**
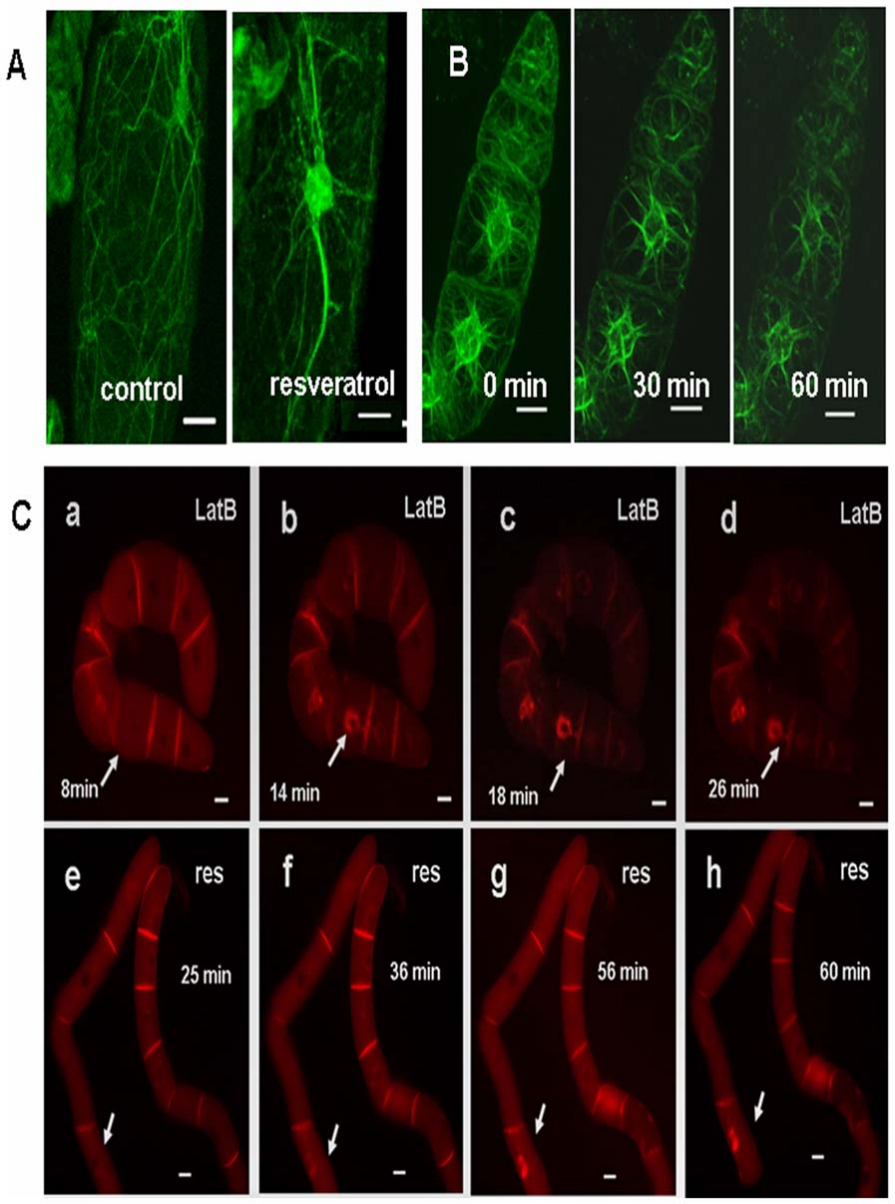
Response of actin filaments to resveratrol. **A** Actin organization in *V. rupestris* in a control cell and after 30 min treatment with 50 µM resveratrol visualized by fluorescent phalloidin. **B** Actin response to 50 µM resveratrol *in vivo* using the actin marker tobacco GFP-11. Size bars = 20 µm. **C** Relocation of the auxin-efflux regulator PIN1-RFP after treatment with the actin inhibitor LatB (2 µM) or with resveratrol (50 µM). Arrows indicate relocalisation of the PIN1-RFP marker. Size bars = 20 µm. All images were captured using an AxioImager Z.1 microscope (Zeiss) equipped with an ApoTome microscope slider through the filter sets 38 HE for FITC or GFP (excitation at 470 nm, beamspliter at 495 nm, and emission at 525 nm) or 43 HE for PIN1-RFP (excitation at 550 nm, beamsplitter at 570 nm, and emission at 605 nm) respectively.

In contrast to microtubules, the response of actin filaments to resveratrol was pronounced in *V. rupestris* (Figure 5A). As compared to untreated controls, where fine strands of actin were observed in the periphery of the cells, actin filaments were strongly bundled and had contracted towards the nucleus 30 min after treatment with resveratrol. Since transgenic grapevine marker lines expressing GFP fusions of cytoskeletal markers are not available for *in vivo* studies, we tested, whether resveratrol was able to induce actin response in the transgenic tobacco BY-2 line GFP-11 [27] expressing the fluorescently tagged FABD-actin marker. Here we could observe that after addition of resveratrol (50 µM) actin filaments were progressively depleted from cell periphery, whereas simultaneously perinuclear bundles of actin appeared within the first 30 min. This actin reorganisation developed progressively over the following time period (Figure 5B).

To functionally verify this resveratrol-induced response of actin bundling, we assessed actin-dependent cellular events. Since alterations of actin organisation interfere with the dynamic localisation of the auxin-efflux component PIN1 [28], we tested the resveratrol response in a transgenic tobacco BY-2 line expressing AtPIN1 in fusion with RFP under control of its own promoter [29]. When actin filaments were eliminated by Latrunculin B, the reintegration of PIN1-RFP into the plasma membrane was affected resulting in intracellular agglomerations (Figure 5C, upper row). Likewise, 50 µM resveratrol were able to induce a similar agglomeration, but with a delay of about 15 min as compared to treatment with Latrunculin B (Figure 5C, lower row). This rapid cellular response to resveratrol was followed, a day later, by a decrease of mitotic index, and a stimulation of cell death (Figure S1, Text S1). These responses were dependent on the concentration of resveratrol and were affected already at the lowest tested concentration of 1 µM resveratrol. In addition, the synchrony of cell division, a diagnostic marker for the activity of actin-dependent auxin transport [28], was progressively disrupted resulting in a progressive decrease of a diagnostic frequency peak of 6-celled over 5-celled files when the concentration of resveratrol reached 10 µM.

These findings show that actin reorganisation, a cellular marker for hypersensitive-reactive cell death, could be triggered by resveratrol as reported earlier for the Harpin elicitor [30]. In contrast, microtubules that are eliminated by the elicitor in *V. rupestris*, do not respond markedly to resveratrol treatment. The actin response to resveratrol is stronger in *V. rupestris*, weaker in cv. ‘Pinot Noir’. Similar to the situation in the *Vitis* cell lines, treatment of BY-2 cells with resveratrol is followed by cell death (Figure S1).

## Discussion

The resistance of wild American *Vitis* species such as *V. rupestris* is correlated with a readily expressed hypersensitive reaction [2]. European cultivated grapevines such as cv. ‘Pinot Noir’ express only basal defence, which becomes manifest in the accumulation of defence-related transcripts [20]. The comparison of the two cell lines should therefore provide insight into differences and overlaps between basal defence and HR. Here, we have shown that the product of StSy, low *trans*-resveratrol and its glycoside *trans*-piceid accumulated in cv. ‘Pinot Noir’, but formed abundant *trans*-resveratrol and the oxidised dimer δ-viniferin in *V. rupestris*. Exogenous resveratrol inhibited cell growth, and activated defence-related responses such as rapid alkalinisation, induction of *PR*5 and *PR*10, actin bundling, and cell death. All of these responses were manifest in both cell lines, but significantly stronger in *V. rupestris*. Both cell lines induced formation of ROS by Harpin elicitor, but resveratrol could trigger ROS-formation only in *V. rupestris*. These observations indicate that resveratrol, in addition to its classical role as phytoalexin, exerts additional roles that seem to be linked with the execution of hypersensitive cell death.

**Figure 6:**
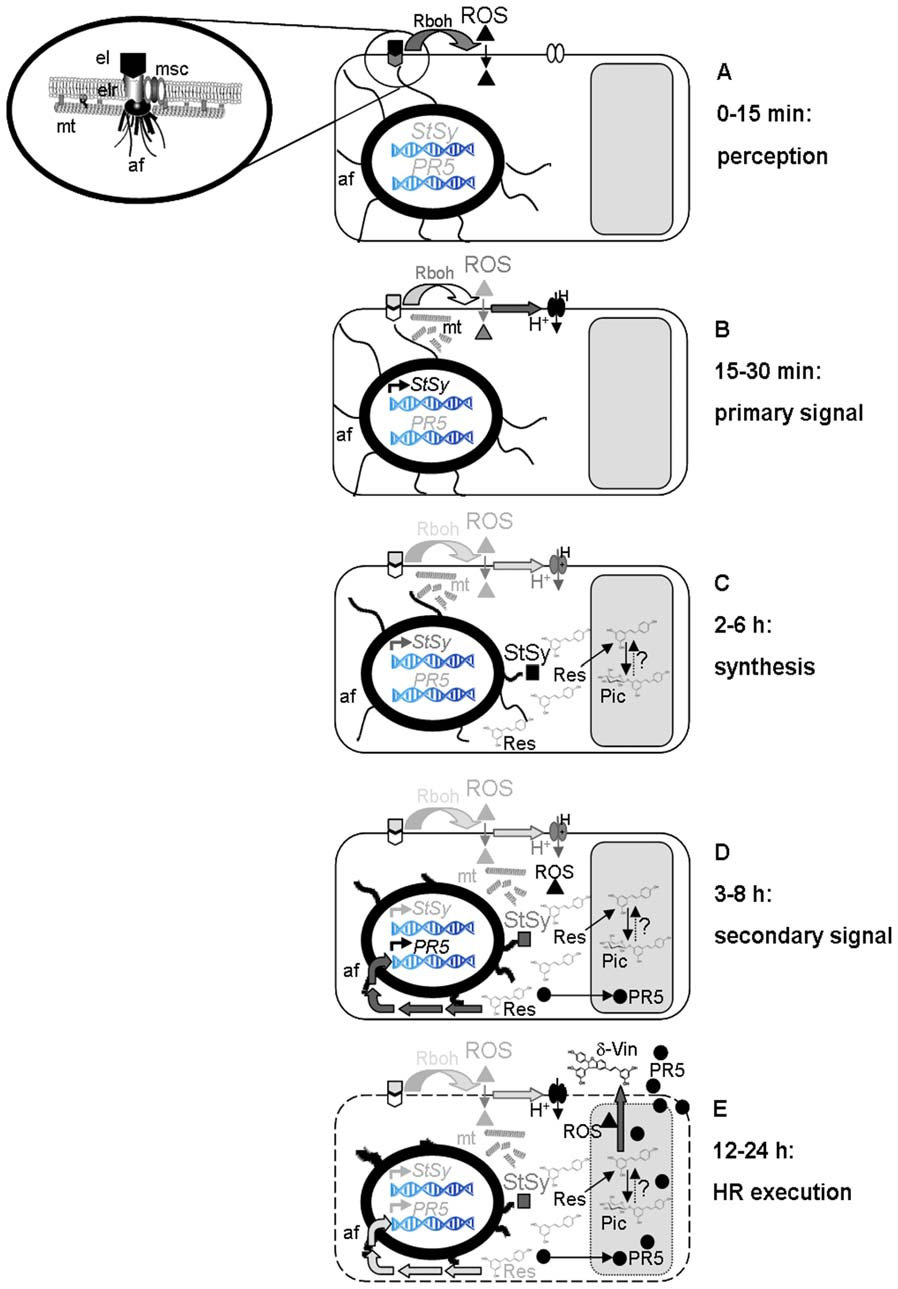
Model for the resveratrol as a secondary signal of elicitor-triggered hypersensitive response in *Vitis* cell. **A** Perception by binding of the elicitor (el) to a receptor (elr) interactimg with a mechanosensitive ion channel (msc) and submembraneous microtubules (mt) and actin filaments (af). Binding activates the NADPH-oxidase Rboh leading to apoplastic reactive oxygen species (ROS), which can permeate into the cytoplasm. **B** A primary signal generated by microtubule disruption activates defence-genes, especially *stilbene synthases* (*StSy*). In parallel, ROS activate proton influx. **C** Synthesis of resveratrol by StSy is accompanied by progressive bundling of actin filaments (heralding commitment for programmed cell death) and partial translocation of resveratrol into the vacuole, where it can be glycosylated into inactive piceid (in cv. ‘Pinot Noir’) or accumulate as aglycon (in *V. rupestris*). **D** Resveratrol as secondary signal initiates accelerates hypersensitive cell death by a second oxidative burst, and induction of *PR*5. In parallel, actin contraction is accentuated. **E** Execution of hypersensitive cell death results in vacuolar breakdown releasing PR5 and resveratrol. Contact of resveratrol with ROS forms the highly cytotoxic δ-viniferin.

Although the cellular responses to the Harpin elicitor are complex, the results from the present work and our previous study [20] allow to sketch down a first working model (Figure 6). The initial step involves perception of Harpin, either by a receptor (Figure 6A), or by membrane-permeabilisation in an ionophore-like manner [30]. Perception initiates signaling including calcium influx [20], probably through a mechanosensitive ion channel [31], [32], and apoplastic oxidative burst. The ROS, are activated within minutes by Harpin in both lines, and within about 30 min by resveratrol (only in *V. rupestris*) are necessary but not sufficient to mediate the response of *StSy* to Harpin (Figure 3). DPI and catalase inhibit Harpin-induced *StSy* induction differently, indicating that different ROS species might trigger different signaling pathways.

Signal perception (Figure 6A) generates a primary signal (Figure 6B) connected with a disruption of microtubules. Besides their classical functions, microtubules participate in the sensing of stimuli [33]. The two cell lines differ in microtubular dynamics manifest as altered abundance of detyrosinated α-tubulin accumulating in stable microtubules [20]. In cv. ‘Pinot Noir’, microtubules were more dynamic and remained intact, in *V. rupestris*, they were more stable and disrupted in response to Harpin. The exact timing of this microtubule response is difficult to determine, since inspection of a cell population stained by immunofluorescence, will detect only advanced stages of disruption obvious at 30 min. To detect earlier stages, it is necessary to follow the response in individual cells over time *in vivo*. We have therefore launched the generation of transgenic *Vitis* expressing fluorescently tagged cytoskeletal markers. Irrespective of the exact timing of the microtubule response, experiments where microtubular disruption in *V. rupestris* was sufficient to trigger accumulation of *StSy*, indicate a stable, detyrosinated, population of microtubules participating in primary defence signaling. A straightforward working hypothesis would propose a microtubule-lever structure that participates in the gating of mechanosensitive calcium channels [31], [34] driving defence signaling. In contrast to Harpin, exogenous resveratrol does not trigger a microtubular response (Figure 4). Parallel to microtubules, actin filaments respond by progressive bundling and contraction towards the nucleus, a response that is very pronounced in *V. rupestris*, but barely detectable in cv. ‘Pinot Noir’ [20]. The function of this actin response will be discussed below.

In parallel to the microtubule response, Harpin induces drastic changes of extracellular alkalinisation [24], inhibited by very low concentrations of Gd^3+^, an inhibitor of mechanosensitive Ca^2+^ channels [20]. Exogenous resveratrol, although inducing alkalinisation, failed to induce *StSy* transcripts (Figure 3), demonstrating that alkalinisation (in contrast to microtubule disruption) was not sufficient to trigger a defence response in grapevine cell system.

The primary signal is followed by the accumulation of *StSy* transcripts at 30 min [20], resveratrol (Figure 6C) from about 2 h after addition of Harpin (Figure 1). As pointed out above, the induction of *StSy* requires ROS. However, H_2_O_2_ was not sufficient to trigger expression of *StSy* transcripts indicating concerted action of parallel, non-redundant pathways, for instance, the mitogen-associated protein kinases (MAPK) mediating calcium-independent Harpin-induced expression of defence genes [30].

Due to its toxicity for the producing cell itself, resveratrol must be either sequestered or secreted. In fact, both mechanisms seem to be at work. In ripening berries that accumulate resveratrol without pathogen challenge, StSy was found in vesicles near the plasma membrane, suggesting secretion into the apoplast [35]. When resveratrol was induced by methyl jasmonate in grapevine cells high amounts accumulated in the vacuole [36].

Resveratrol shows antifungal activity against grapevine pathogens and thus meets the criteria for a phytoalexin, but resveratrol seems to be more than a final product. (Figure 6D). Resveratrol can be further metabolised into oxidative δ-viniferin that is even more effective against *P. viticola* zoospores or into the ineffective glycoside piceid [12], [37]. However, all stilbenes show not same toxicity against pathogen. Resveratrol had real inhibitory effects on conidial germination of *B. cinerea* liquid cultures when used at concentration ranging from 60 µg/ml (25% inhibition) to 160 µg/ml (100% inhibition), i.e., from 2.6 to 7×10^−4^ M [22]. Piceid has never shown any toxic activity against *P.viticola* zoospores, even at concentration >100 µM [12], whereas viniferin has an antifungal activity upon germination of *B.cinerea* conidia (ED50 = 36 µg/ml, 7.9×10^−5^ M) similar to that of pterostilbene, which is the most toxic stilbene [37]. The differential conversion of resveratrol in *V. rupestris* versus cv. ‘Pinot Noir’ may be a branching point between basal immunity and HR. Our results are supported by circumstantial evidence from molecular farming of the therapeutically interesting resveratrol. Here, induction of stilbene synthesis by methyl jasmonate accumulated mostly piceid in *V. vinifera* [38], but large quantities of resveratrol and viniferin in a hybrid with the HR-competent American *V. berlandieri* [36].

These observations shift resveratrol-metabolising enzymes into the focus. Glycosylation into piceid might be triggered. In contrast, resveratrol-oxidising basic peroxidase isoenzymes [39], [40] might be of particular interest as regulatory targets, because they are differentially localised either in the apoplast (isoenzyme A1, B3) or the vacuole (isoenzyme B5), and have been associated with constitutive defence of grapevine against fungi [41]. A key role of resveratrol metabolisation is also supported by the fact that resveratrol could be identified as a target of fungal effectors. Fungal laccases of *Botrytis cinerea* cause an oxidative degradation of resveratrol [42] allowing the fungus to escape from the action of grapevine phytoalexins [43]. If resveratrol metabolism acts as a switch between different types of immunity, selective pressure on coevolving pathogens would be expected to favour effectors targeted to this developmental switch.

When resveratrol and its derivatives act as secondary signals, specific resveratrol responses must exist. We observed that resveratrol stimulated oxidative burst, reorganisation of the actin cytoskeleton, and induction of defence genes such as *PR*5. Despite a certain overlap with Harpin-triggered responses, specific differences exist:

- Harpin caused disintegration of microtubules, resveratrol failed to do so, even in the highly responsive *V. rupestris* (Figure 4).
- Oxidative burst in response to Harpin was detected already at 5 min even in cv. ‘Pinot Noir’ (Figure 3), but not earlier than 30 min in response to resveratrol, even in *V. rupestris*. Moreover, alkalinisation responded slowly to exogenous resveratrol (Figure 2), but rapidly to Harpin [20]. The shift in timing (about 30 min) would be consistent with a model, where the proton channel was activated by the resveratrol-triggered oxidative burst.
- The pattern of gene expression differed. Whereas Harpin triggered a rapid, but transient response of *StSy* and *RS* (30 min, peak at 2 h), these genes did not respond to resveratrol. Instead, resveratrol triggered a slower, but sustained response of *PR*10, and, prominently, of the osmotin-type *PR*5 (Figure 2). *PR*10 was also among the genes tested for their response to Harpin [20] and was found to accumulate from about 2 h (but exclusively in *V. rupestris*, not in cv ‘Pinot Noir’) – a temporal pattern consistent with a mechanism, where the resveratrol generated by the Harpin-induced *StSy*/*RS* triggers a second, delayed, but sustained wave of gene expression.

The biological role of these secondary signals (second oxidative burst, synthesis of osmotin-type PR5, progressive actin bundling) seems to unfold during the execution of hypersensitive cell death (Figure 6E): The oxidative burst generated by resveratrol could be used by peroxidases in apoplast and vacuole [44] to convert resveratrol into the highly potent oxidative oligomers, as shown for a HR-like response triggered in grapevine by an elicitor from *Trichoderma viride* [45]. Resveratrol would, thus, trigger a response that drives its own conversion towards the more potent viniferins representing the actual phytoalexins. Additionally, resveratrol-triggered ROS might further activate downstream signal reactions such as defence-related gene expression and HR [3]. Interestingly, resveratrol failed to induce an oxidative burst in cv. ‘Pinot Noir’, i.e. the two cell lines differ in their competence for resveratrol-dependent oxidative burst, which means that the generation of ROS is not a molecular property of resveratrol *per se*.

PR5 protein belongs to a widely distributed group of defence proteins sharing sequence similarity with the intensely sweet protein, thaumatin [46], which were proved to inhibit the development of fungal pathogens, probably by binding fungal 1,3-β-D-glucans [47], but also can stimulate phytoalexin accumulation in *Arabidopsis thaliana* [48]. In this study, the resveratrol-responsive PR5 protein belongs to osmotin-like subset of these proteins [49], contains an N-terminal signal peptide, but no ER-retention signal suggesting secretion or transport into the vacuole. Irrespective of its exact localisation, the induction of PR5 by resveratrol proceeds to a very similar extent in both grapevine cell lines, i.e. in contrast to the resveratrol-triggered oxidative burst there seems to be no difference in competence for triggering PR5.

The progressive bundling of actin initiates earlier than the other two responses and is observed in response to both Harpin [20] and resveratrol (Figure 5). It can be followed *in vivo* in BY-2 cells using GFP-tagged actin-marker lines (GFP-11) after Harpin and resveratrol (Figure 5) treatment. Reorganisation of actin is common in plant defence and has been traditionally interpreted in the context of actin-dependent transport of secretory products to the infection site and local activation of callose synthesis [49]. Exogenous resveratrol affected the polar localization of the auxin-efflux component PIN1 (Figure 5) similarly to Latrunculin B, consistent with actin-dependent recycling of PIN-proteins between plasma membrane and endosomal compartments [50]. Resveratrol-triggered bundling of actin should therefore affect auxin transport. In fact, cell division synchrony, a highly sensitive reporter for auxin transport [51] was affected by resveratrol in tobacco BY-2 wild type cell line (Figure S1, Text S1). However, bundling of actin filaments also represents an evolutionary conserved element of programmed cell death [26]. Electrical detachment of submembraneous actin by nanosecond pulses can trigger actin contraction, loss of membrane integrity, and programmed cell death in the absence of pathogens or elicitors [52], [53]. The bundling of actin triggered by Harpin and resveratrol has therefore to be seen in the context of a developmental program that culminates in loss of membrane integrity and thus mediates in the execution of cell death. It should be noted that actin bundling initiates earlier than the significant quantity of resveratrol has been synthetised and therefore must be controlled by a different pathway – probably at the membrane-cytoskeleton interface (Figure 6A). However, the response might be potentiated by resveratrol.

As a result of these three mechanisms triggered by resveratrol, highly toxic oxidative products (δ-viniferin) are produced, proteins that can attack fungal cell walls (PR5) accumulate, and the (programmed) loss actin-dependent membrane integrity is potentiated. This will culminate in the final blow: vacuolar breakdown and release of toxic phytoalexins and PR5 contributing to the efficient and active of HR-competent host cells to pathogenic invaders.

Interestingly, in addition to its function as a signaling regulator of HR cell death above, resveratrol is also well-known due to its pharmacological effects on human health. Recently, autophagy, a self-destructive mechanism, by which eukaryotic cells clear damaged proteins and organelles, remobilise of cell contents and maintain energetic requirements, has been considered conferring longevity under nutrient limitation [54]. By activation of SIRT-1, a NAD+-dependent deacetylase, resveratrol not only induces autophagy and extends lifespan, but also suppresses colitis and colon cancer [15], [54], [55], [56]. Inhibition of S6 kinase by resveratrol also suppresses the starvation-induced autophagy [57], [58]. Two alternative pathways may act in concert or in parallel to exert the anti-aging effect of resveratrol. These findings raise a question: Why can antimicrobial molecules produced by plants benefit to human health? Obviously, the underlying mechanism is unlikely explained by a fortuitious coincidence. One possibility is called “common origin hypothesis” that animals and plants share the common ancestor in the biosynthetic pathway, or, since animals have lost the ability to synthesize certain polyphenols, but they have retained the ability to be activated by these molecules [56], [59]. Another popular explanation is, according to xenohormesis hypothesis proposed by Howitz and Sinclair [17], organisms have evolved the ability to sense stress-induced molecules i.e. polyphenols from other species in their environment, and used the cues to prepare in advance for loss of food supply and adversity [60].

In conclusion, the comparison of the two grapevine cell lines has uncovered that resveratrol functions as a secondary signaling molecule, contrast with its normal phytoalexin role, to regulate the key switch of the hypersensitive cell death in *Vitis* resistance. This leads new questions: What are the direct targets of resveratrol in plant defence signaling? Why can resveratrol trigger oxidative burst in *V. rupestris*, but not in cv. ‘Pinot Noir’? Is actin-dependent membrane stability involved in the signaling preparing a cell for the “final call” to undergo programmed cell death in response to resveratrol? Is PR5 simply a component of basal defence? Last, but not least: what are the receptors that trigger basal defence and/or HR?

## Materials and Methods

### Cell culture

Cell lines for *Vitis rupestris* and *Vitis vinifera* cv. ‘Pinot noir’ generated from leaves were cultivated as described in Qiao *et al.* [20]. The transgenic tobacco (*Nicotiana tabacum* L. cv. Bright Yellow 2) cell line BY-2 GFP-11 [27] stably expressing the actin marker Fimbrin Actin-Binding Domain 2 (FABD2) fused with GFP was cultivated in presence of 30 mg l^−1^ hygromycin. The tobacco cell line PIN1-RFP [29] expressed stably the auxin-efflux regulator AtPIN1 in fusion with RFP under control of the *AtPin1* promoter was cultivated in addition with 100 mg l^−1^ kanamycin.

The Harpin elicitor (Messenger, EDEN Bioscience Corporation, Washington, USA; 3% active ingredient Harpin protein) was dissolved in water to yield a stock solution of 300 mg.ml^−1^. Resveratrol (Sigma-Aldrich, Deisenhofen, Germany) was dissolved in absolute ethanol to a stock solution of 100 mM. Diphenylene-iodonium chloride (DPI) and Latrunculin B (Lat B) were purchased from Sigma-Aldrich, Deisenhofen in Germany and prepared in dimethylsulfoxide (DMSO) to stock solution of 10 mM and 1 mM respectively. Hydrogen peroxide [H_2_O_2_, Sigma-Aldrich, 30% (w/w) in water] was diluted with water to a stock solution of 10 mM. Catalase was dissolved in 50 mM Tris-HCl, pH 7.0 to obtain a stock solution of 100 U.µl^−1^. All treatments were accompanied by solvent controls, where the maximal concentration of solvent used in the test samples was administered.

### Dose-response of cell growth and extracellular alkalinisation over resveratrol

Cell growth was quantified by measuring packed cell volume (PCV) at 7 days after subcultivation with or without the presence of different concentrations of resveratrol and equal volumes of solvent ethanol [61]. Time courses of growth inhibition were also performed. In parallel, mortality was assessed using Evan's Blue [62]. The experiment was repeated four times. Extracellular alkalinisation was evaluated according to Qiao *et al.* [20]. The course of pH changes was plotted over time. Dose-response curves were obtained by plotting the maximal change of pH over resveratrol concentration.

### Expression analysis

To evaluate the effect of exogenous resveratrol on the transcription of defence-related genes, 1 ml cells (5 d) were treated with 50 µM resveratrol or ethanol as a control for indicated time points (0.5, 1 or 3 h). Transcripts of genes encoding resveratrol synthase (RS), stilbene synthase (StSy), pathogenesis-related proteins 5 and 10 (PR5, PR10), and polygalacturonase inhibiting protein (PGIP) were quantified by semi-quantified reversible transcription PCR (RT-PCR) using the primer combinations defined in Kortekamp [63]. PCR products were quantified using the Image J software (http://rsbweb.nih.gov/ij/) and standardized relative to elongation factor 1-α as internal standard [64]. The results were plotted as fold increase of transcript abundance as compared with the untreated control. The data represent the mean ± standard errors from at least three independent experimental series.

To determine the influence of reactive oxygen species (ROS) on the expression of the marker gene *StSy*, 1 ml cells was induced for 2 h in the presence of different combinations of the elicitor Harpin (9 µg l^−1^), H_2_O_2_ as ROS donor (10 µM), the NADPH oxidase inhibitor DPI (10 µM) or the ROS scavenger catalase (100 U). Experiments were performed three biologically repeats.

### Fluorescent detection of ROS

To examine production of ROS induced by Harpin or resveratrol, 200 µl cells (at day 3 to 4 after subcultivation) were suspended into 800 µl PBS, preequilibrated on a shaker for at 1 h and then supplemented with dihydrorhodamine 123 (DHR 123, final concentration 10 µM), a cell-permeable fluorogenic probe reporting oxidative burst [65]. After 30 min incubation, cells were washed 3 times using pre-warmed PBS at 37°C and resuspended in 1 ml PBS in combination with 50 µM resveratrol, or with 9 µg l^−1^ Harpin as positive control, or with the solvent ethanol as negative control. Changes of the fluorescent signal were followed over time under an AxioImager Z.1 microscope (Zeiss, Jena, Germany) using a the filter set 38 HE (excitation at 470 nm, beamsplitter at 495 nm, and emission at 525 nm), a 40× objective and a constant exposure time of 300 ms. Production of ROS fluorescence was quantified by Image J software (http://rsbweb.nih.gov/ij/).

### Cytoskeletal visualisation in *Vitis* cells

The response of the cytoskeleton was visualized as described previously [19] in fully expanded cells 10 days after subcultivation and treatment with either the solvent control, or 50 µM resveratrol, or 9 µg l^−1^ Harpin for 30 min, respectively. Cells were observed under a confocal laser scanning microscope (TCS SP1; Leica, Bensheim, Germany) using ×63 oil immersion objective with excitation by the 488 nm laser line of the ArKr laser and a four-frame averaging protocol.

### *In vivo* observation of resveratrol responses in transgenic BY2 cell lines

For *in vivo* observation of cellular responses to resveratrol, 200 µl aliquots of GFP-11 cells were collected at day 4 and diluted into 800 µl MS liquid medium supplemented with 50 µM resveratrol and then immediately examined. The localisation of PIN-RFP [28] was followed after treatment with either Latrunculin B (final concentration 2 µM) or resveratrol (final concentration 50 µM) over time. All time series were recorded under an AxioImager Z.1 microscope (Zeiss) equipped with an ApoTome microscope slider through the filter sets 38 HE (excitation at 470 nm, beamsplitter at 495 nm, and emission at 525 nm) for GFP imaging, and 43 HE (excitation at 550 nm, beamsplitter at 570 nm, and emission at 605 nm) for RFP imaging. All images were processed and analysed using the AxioVision software (Rel. 4.5; Zeiss) as described earlier [66].

### Quantification of stilbenes

After treatment with Harpin (9 µg l^−1^), cells were collected at indicated time points (0, 2, 4, 6, 8, 10, 24 or 48 h) by centrifugation (5 000 rpm, 5 min). Stilbenes were extracted according to Tassoni *et al.* [4] with minor modifications. 3 g fresh weight of cells was mixed with 20 ml methanol and homogenised by an ultrasonic processor (UP100H, Hielscher, Germany) for 3 min. The homogenate was incubated for 1 h in the dark at room temperature on a rotatory shaker at 150 rpm and filtered through filter paper (Whatman®, Schleicher & Schüll, Germany). The filtrate was concentrated to a residual volume of 5 ml in a glass tube at 40°C (Heating Bath B490, BÜCHI, Germany) at 280 rpm (Rotavapor R-205, BÜCHI, Germany) and a pressure of 80 Pa (Vacuubrand CVC2, Brand, Germany). Stilbenes were isolated from the aqueous phase by adding 2 ml 5% (w/v) sodium bicarbonate to buffer pH, and three aliquots of 5 ml ethyl acetate. The pooled ethyl acetate phase was completely dried and the residue suspended in 2 ml methanol prior to injection into the HPLC.

Stilbenes were analysed using a high performance liquid chromatograph, HPLC (Agilent, 1200 series, Waldbronn, Germany) equipped with a Phenomenex Synergi hydro RP column (150×4.6 mm, particle size 4 µm, Phenomenex; Aschaffenburg, Germany), a DAD detector, and a quaternary valve. The flow rate was 0.8 ml min^−1^, and the injection volume is 20 µl. The mobile phases included acetonitrile (ACN), methanol and water in the following gradient: 2 min ACN/water (10/90 v/v); 15 min ACN/water (40/60 v/v); 30 min ACN/methanol (50/50 v/v); 32 min ACN/methanol (5/95 v/v); 35 min ACN/methanol (5/95 v/v); 39 min ACN/water (10/90 v/v); 42 min ACN/water (10/90 v/v).*Trans*-resveratrol, *trans*-piceid, and δ-viniferin were quantified and identified using an external standard on the basis of retention time and UV-VIS spectra. The standards for *trans*-resveratrol, *trans*-piceid (Phytolab, Vestenbergsgreuth, Germany) and δ-viniferin were dissolved in methanol at a concentration of 100 mg l^−1^ respectively. Calibration curves determined using these standards were linear (r^2^>0.99) and used for quantification of the samples. At least five independent experimental series were conducted.

## Supporting Information

Figure S1 **Dose-dependent cellular responses of tobacco BY-2 wild type cell to resveratrol treatment.**
(DOC)

Text S1 **Description of cell pattern experiments of tobacco BY-2 wild type.**
(DOC)
