# Supplementary material for: The Phytoalexin Resveratrol Regulates the Initiation of Hypersensitive Cell Death in Vitis Cell
Source: PLoS One. 2011 Oct 28;6(10):e26405. doi: 10.1371/journal.pone.0026405 (PMC3203900; doi:10.1371/journal.pone.0026405)
Supplement: Figure S1 — Dose-dependent cellular responses of tobacco BY-2 wild type cell to resveratrol treatment. (DOC) [file pone.0026405.s001.doc]

**Figure S1**. **Dose-dependent cellular responses of tobacco BY-2 wild type cell to resveratrol treatment.**

**A.** Frequency distribution over cell number per file at day 4 after inoculation in presence of different concentration of resveratrol. Error bars indicate standard errors. **B.** Mitotic index over time after subcultivation. Each point represents the mean from 500 scored cells. **C.** The percent of cell death at day 1, 2, 3 after resveratrol treatment. 3000 cells were scored in three dependent experiments to obtain standard errors.


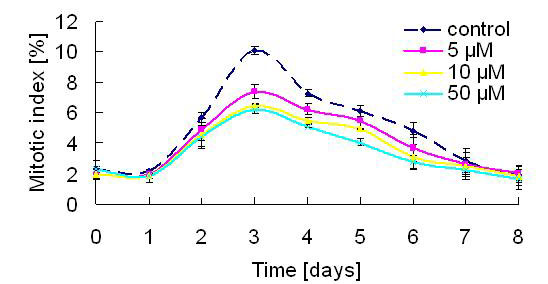


**A**


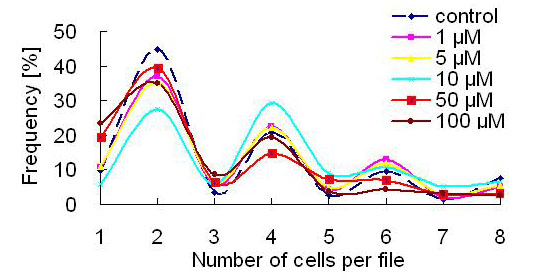


**B**

**A**

**A**


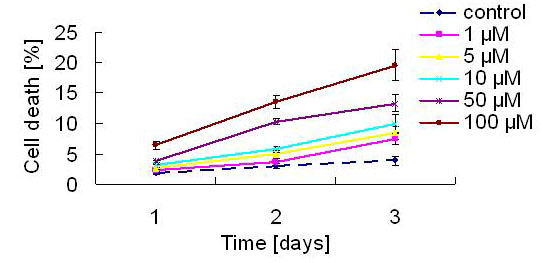


**C**
