# Supplementary material for: The Phytoalexin Resveratrol Regulates the Initiation of Hypersensitive Cell Death in Vitis Cell
Source: PLoS One. 2011 Oct 28;6(10):e26405. doi: 10.1371/journal.pone.0026405 (PMC3203900; doi:10.1371/journal.pone.0026405)
Supplement: Text S1 — Description of cell pattern experiments of tobacco BY-2 wild type. (DOC) [file pone.0026405.s002.doc]

**Text S1. Experiment description of cell pattern and cell death of tobacco BY-2 wild type**

For synchronized cell division pattern, 1 ml aliquots of BY-2 cells were collected at 4 days after inoculation with different concentration of resveratrol or absolute ethanol as a control. Then, cells were immediately viewed under the microscope above. The frequency distribution over the number of cells per individual file was constructed from 500 individual files (containing up to 8 cells per file).The data was repeated three times to obtain an standard error bar.

For the mitotic indices, 0.5 ml aliquots of cell suspension were fixed in Carnoy fixative [96% (v/v) ethanol: acetic acid, 3:1 (v/v)] complemented by 0.5% (v/v) Triton X-100. After washing three times with PBS buffer, cells were stained with 2´-(4-hydroxyphenyl)-5-(4-methyl-1-piperazinyl)-2,5´-bi (1H-benzimidazole) trihydrochloride (Hoechst 33258, Sigma-Aldrich, ﬁnal concentration 10 ng ml^-1^). Samples were investigated with an AxioImager Z.1 microscope (Zeiss) using the filter set 49 (excitation at 365 nm, beamsplitter at 395 nm, and emission at 445 nm). The mitotic indices were calculated as the number of cells in mitosis divided by the total number of cells counted. For each time point, 1 000 cells were scored.

To analyse the cell viability, BY-2 wt cells was stained by 2.5% Evans blue dye and visualized under an AxioImager Z.1 microscrope (Zeiss) under the bright field. The percent of cell death was calculated by the ratio of the number of dead cells to the number of total cells. 3 000 cells were scored in three independent experiments. Error bars indicate ± standard error.
